# Sleep and Stroke: Opening Our Eyes to Current Knowledge of a Key Relationship

**DOI:** 10.1007/s11910-022-01234-2

**Published:** 2022-10-03

**Authors:** Valerio Brunetti, Eleonora Rollo, Aldobrando Broccolini, Giovanni Frisullo, Irene Scala, Giacomo Della Marca

**Affiliations:** 1 UOC Di Neurologia, Dipartimento Scienze Dell’Invecchiamento, Neurologiche, Ortopediche E Della Testa-Collo, Fondazione Policlinico Universitario A. Gemelli IRCCS, Largo A. Gemelli 8, 00168 Rome, Italy; 2 Department of Neurosciences, Università Cattolica del Sacro Cuore, Rome, Italy

**Keywords:** Sleep, Stroke, Sleep apnea, Insomnia, Restless legs syndrome, Circadian rhythm

## Abstract

**Purpose of Review:**

To elucidate the interconnection between sleep and stroke.

**Recent Findings:**

Growing data support a bidirectional relationship between stroke and sleep. In particular, there is strong evidence that sleep-disordered breathing plays a pivotal role as risk factor and concur to worsening functional outcome. Conversely, for others sleep disorders (e.g., insomnia, restless legs syndrome, periodic limb movements of sleep, REM sleep behavior disorder), the evidence is weak. Moreover, sleep disturbances are highly prevalent also in chronic stroke and concur to worsening quality of life of patients.

Promising novel technologies will probably allow, in a near future, to guarantee a screening of commonest sleep disturbances in a larger proportion of patients with stroke.

**Summary:**

Sleep assessment and management should enter in the routinary evaluation of stroke patients, of both acute and chronic phase. Future research should focus on the efficacy of specific sleep intervention as a therapeutic option for stroke patients.

## Introduction

Sleep is a basic human need, essential for physical and mental health. Stroke is a leading cause of death and disability worldwide. A close relationship between sleep and stroke has been largely recognized [1••]. Several sleep disturbances have been studied as risk factors for stroke, including sleep-disordered breathing (SDB) [2], insomnia [3], restless legs syndrome (RLS) [4], periodic limb movements of sleep (PLMS) [4], REM sleep behavior disorder (RBD) [5], narcolepsy [6], circadian rhythm disorders [7•], and short and long sleep duration [8•]. Moreover, sleep is severely disrupted in patients with stroke [9] in both acute and chronic phase, and stroke patients experience sleep-dependent changes of physiological functions [10]. Sleep has a well-established role in synaptic plasticity [11]. Therefore, post-stroke sleep disruption may interfere with synaptic plasticity and with brain extracellular waste removal, that are essentials for stroke recovery [12]. As a matter of fact, in 2020, a task force of European experts in neurology, stroke, respiratory medicine, and sleep medicine proposed shared guidelines for the management of sleep disorders in patients with stroke [13••].

**Fig. 1:**
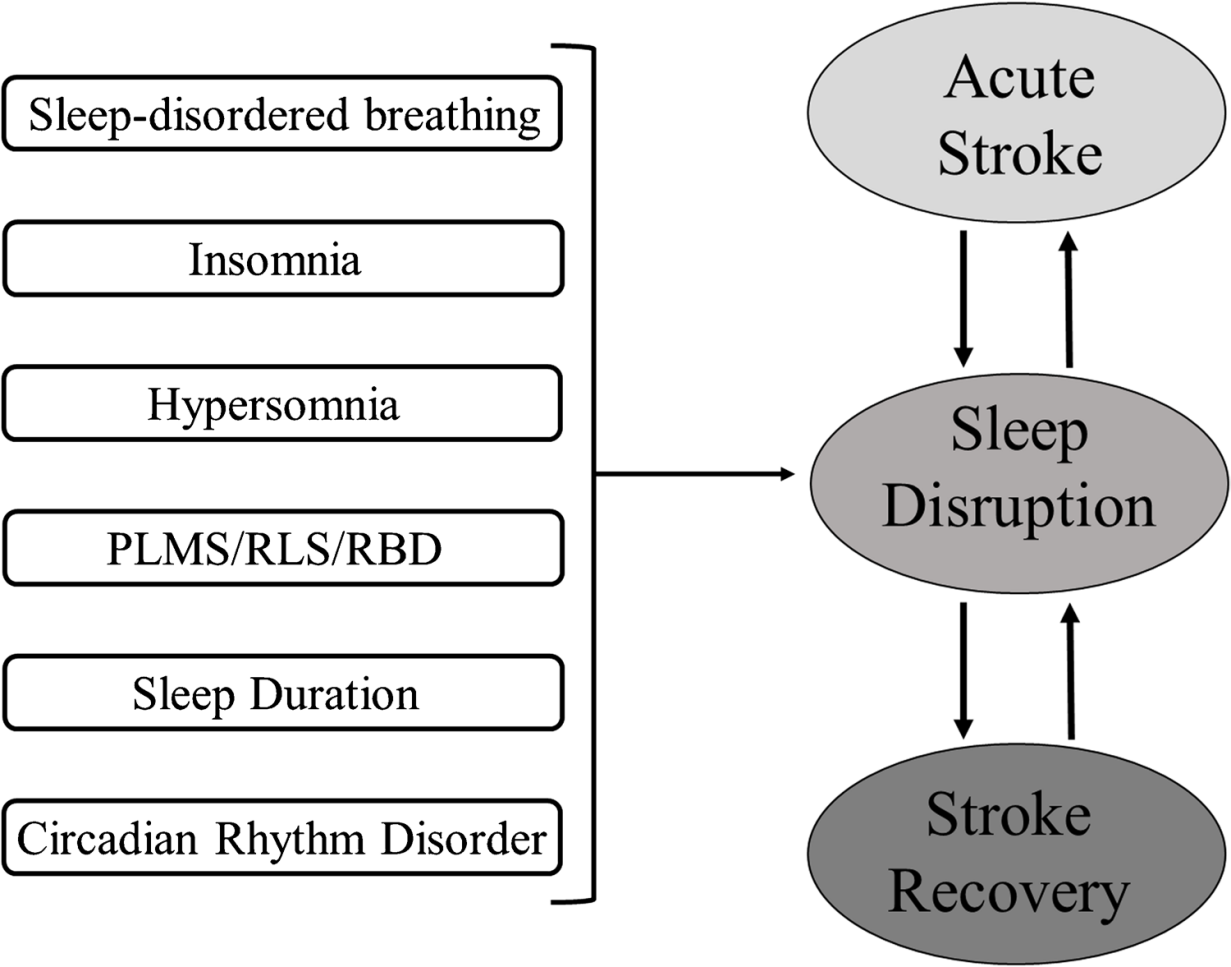
Sleep and its disorders strictly interconnected with stroke

In the current paper, we will review how sleep and its disorders are strictly interconnected with stroke, playing a pivotal role as a stroke risk factor, appearing de novo as consequence of stroke, and modifying the course of the acute and chronic stroke phase (see Fig. 1).

## Sleep Apnea and Stroke

Sleep apnea (SA) is the most extensively studied SDB in association with stroke [14]. SA is characterized by repetitive and intermittent cessation of airflow and encompasses two main phenotypes: obstructive SA (OSA), due to increased upper airways resistance, and central SA (CSA), due to lack of the respiratory drive.

SA, and particularly OSA, is one of the most common comorbidities in patients with stroke, acting as an independent risk factor [15], being high prevalent in the acute stroke phase [2], and playing a pivotal role in stroke recovery [16•] and stroke recurrence [17].

### Sleep Apnea as Risk Factor for Stroke

OSA is a high prevalent in general population and is a potential modifiable risk factor for stroke. The hazard ratio of the risk of stroke in OSA population ranges from 1.97 [15] up to 4.63 [18]. OSA increases the risk of stroke through direct and indirect pathways. Among direct mechanisms, OSA leads to repetitive intermittent hypoxia, increased sympathetic activity, cerebral hemodynamic changes, hypercoagulability, endothelium dysfunction, and to an increased inflammatory response [19•, 20]. Moreover, OSA indirectly prompts stroke concurring to arterial hypertension and atrial fibrillation [21, 22].

The risk of stroke is directly related to OSA severity [23]; however, a definite phenotype of OSA, such as biomarkers predictive of cerebrovascular injury, is still missing. Recent evidence suggests that incidence of stroke is significantly higher in patients with OSA showing excessive daytime sleepiness (EDS) [24, 25]. Conversely, another recent research did not show any association between OSA symptoms and risk of cardiovascular diseases [26], but identified hypoxic burden, defined as the area under the desaturation curve associated with respiratory events, as a promising marker of cardiovascular risk in OSA population [26, 27]. From this point of view, new polygraphic metrics behind the number of apneas and hypopneas per hour of sleep (Apnea–Hypopnea Index, AHI) have been recently investigated (e.g., hypoxic burden [26, 27], hypoxia load [28], oximeter-derived pulse rate variability [29], and sleep breathing impairment index [30]) in order to identify markers of cerebrovascular risk in patients with OSA.

For what concerns CSA as risk factor for stroke, any convincing evidence is missing. Only one study on a large cohort of elderly patients showed that CSA and its severity were independent risk factors for stroke [31]. However, since CSA is frequently associated with atrial fibrillation and heart failure, it is possible that more than a specific risk factor, CSA represents a biomarker of other established risk factors for stroke.

### Sleep Apnea in Stroke Patients: New Onset or Pre-existing Condition

Since SA is highly prevalent in patients with stroke, it is debated whether SA is a pre-existing condition or a consequence of stroke. In a recent meta-analysis performed by Seiler et al. [2] conducted on 86 studies for a total of 7096 patients, the prevalence of SA in patients with acute stroke or TIA ranged between 30% (with an AHI > 30/h, consistent with severe SDB) up to 71% (with an AHI > 5/h, consistent with mild SDB); only a slightly lower prevalence was observed in the chronic phase. However, most of the studies included in the meta-analysis evaluated the acute phase, while only few studies [32–37] evaluated the intra-individual evolution from acute to the chronic phase. Moreover, few studies [33–36, 38] evaluated the evolution of SDB in stroke by means of polysomnography (PSG), the standard diagnostic test for SDB in stroke patients [39], reporting an improvement, but not a resolution, from acute to sub-acute/chronic phase, of the SDB. These data were further confirmed by a meta-analysis conducted by Hasan et al. who evaluated the dynamic prevalence of sleep disorders after stroke or TIA [1••]. Recently, the SAS Care 1 study [35] evaluated longitudinally the progression of SDB in a large cohort patient with ischemic stroke or TIA by means of PSG: the authors observed a significant reduction of AHI (baseline: 21 events/h; at 3 months: 18 events/h) driven by a reduction of both the obstructive and central component. Nevertheless, the prevalence of SBD was similar at baseline (85.6%) and at 3 months (82.7%), and a reduction was observed exclusively for severe SBD (AHI > 30/h) and for CSA. Taken together, these data support the hypothesis that OSA may be a pre-existing condition aggravated by stroke, while CSA may appear de novo being a symptom of the acute phase.

Notably, the high prevalence of SDB in stroke is mainly determined by OSA, being the reported prevalence of CSA approximately 12% [2]. In a recent study conducted on a large population of stroke patients tested with a home sleep apnea test [40], the prevalence of CSA was even lower (1.4%). However, the prevalence of CSA in stroke patients is probably underestimated. In fact, CSA is diagnosed exclusively when more than 50% of total apneic events are scored as central. Furthermore, most of the studies evaluating the prevalence of SDB did not score central hypopnea [40]. Since the pathogenic pathways behind OSA and CSA are different, future studies should count separately the AHI of central and obstructive events and consider a mixed respiratory pattern when these two conditions coexist. In fact, studies that took into the account a mixed pattern, characterized by the coexistence of central and obstructive events, observed a high proportion of such pattern in both acute [41] and chronic phases [42•].

Several mechanisms can contribute to exacerbate or aggravate a pre-existing SDB in patients suffering of stroke. Though several studies failed to demonstrate a direct correlation among stroke topography and SDB [43], strokes involving the central respiratory pattern generator can induce respiratory instability and, in turn, promote both central and obstructive SDB [36, 42•]. In the acute stroke, several factors compromise the patency of the upper airways, such as a weakness or incoordination of the pharyngeal [44, 45], intercostal and diaphragmatic muscles [46], increased rostral fluid shift [47], and prolonged supine position [48]. Moreover, a reduced arousal response may concur to increase the length of apnea and the hypoxic burden. Taken together, these data suggest that stroke patients may present a peculiar SDB phenotype characterized by a multifactorial pathogenic mechanism [49], the coexistence of central and obstructive apnea, specific polygraphic features [50, 51], and different cardiovascular risk [52].

### Sleep Apnea and Stroke Outcome

SDB negatively impacts on stroke outcome. In the BASIC [16•] and SAS Care 1 [35] studies, stroke patients with concomitant SDB showed poorer functional outcome at 3 months. Also, during acute phase, patients with SDB are at higher risk of early neurological deterioration [53]. It is supposed that sustained nocturnal hypoxia may play a detrimental role in extension of the ischemic penumbra [54], and that sleep fragmentation may interfere with synaptic plasticity [12]. Still, stroke patients with SDB exhibit a higher risk of stroke recurrence, and the recurrence risk is directly related to SDB severity [17]; the risk of recurrence appears to be linked to OSA rather than CSA [55]. Conversely, CSA, together with nocturnal hypoxia, may predict mortality [55]. Notably, most of studies evaluating the impact of OSA on the stroke outcome were conducted in pre-thrombectomy era. In fact, in last years, stroke outcome significantly improved due to the introduction of endovascular treatment [56]. A recent study conducted on a large stroke registry [57•] reported that stroke patients undergoing to thrombectomy and with a pre-existing diagnosis of OSA showed lower mortality and lower risk of intracranial hemorrhages compared to patients without OSA; the authors speculated that the repeated hypoxic/hypercapnic episodes that occur during sleep in OSA induce neuroprotective adaptations in the brain, increasing the tolerance to the hypoxia/ischemia [58, 59]. Although this study has several limitations, the main one being that OSA is probably largely under-recognized in the examined sample—it pinpoints that new evidence of the role of SDB and, eventually, its treatment are needed in the era of endovascular treatment.

### Diagnosis of Sleep Apnea in Stroke Patients

Notably, in stroke patients’ signs and symptoms of OSA are not predictive [60]. Therefore, in the light of the high prevalence of SA in stroke patients, the search of sleep apnea should be part of routinary stroke care and, ideally, all stroke patients’ should undergo to an instrumental sleep assessment. Conversely, only a minority of stroke patients undergo to a formal sleep evaluation [61•]. Current guidelines of American Academy of Sleep Medicine recommend full-night PSG for screening and diagnosis of SDB in patients with stroke [39]. However, PSG is cost and time expensive, poorly available outside specialized sleep centers, and difficult to perform in acute setting. In the SLEAP SMART trial, home sleep apnea testing performed by means of respiratory polygraphy showed good feasibility and good diagnostic value compared to in-laboratory PSG [62•]. Therefore, in an acute setting, a sleep study with limited-channel devices is a reasonable alternative to PSG, keeping the latter study dedicated to ambiguous cases and to patients showing peculiar comorbidities (i.e., heart failure, chronic obstructive pulmonary diseases, and concomitant sleep disorders) [2, 14, 63].

Since performing a sleep study in all stroke patients is compelling, current research is focusing on identifying predictors of sleep apnea in this population applying promising artificial intelligent techniques [64•, 65, 66], new scores [67], or alternative recording techniques [68].

### Treatment of Sleep Apnea in Stroke Patients

To date, studies evaluating the efficacy of SDB treatment in primary stroke prevention yielded conflicting results. Two recent large trials, the SAVE [69] and the ISAACC [70], failed to demonstrate the effectiveness of continuous positive airway pressure (CPAP) treatment for prevention of cardiovascular events, including stroke. Notably, in a subgroup analysis of SAVE trial, patients that reported a good adherence (> 4 h/night) showed a reduced risk of cerebrovascular events. Similarly, a recent meta-analysis including 13 studies (nine RCTs and four observational studies) reported an efficacy of CPAP treatment in reducing the risk of stroke in patients with good adherence and patients with moderate to severe OSA [71•]. This data was further confirmed by a retrospective cohort study conducted on 5757 Medicare beneficiaries aged ≥ 65 years that revealed that CPAP adherence was associated with a 2% reduction in risk of stroke for each month [72].

To date, studies that evaluated the effectiveness of positive airway pressure (PAP) treatment on neurological outcome and stroke recurrence in patients with stroke yielded conflicting results [73–76]. For a detailed review on studies evaluating the efficacy of SA treatment in patients with stroke, see Boulos et al. [77]. Nonetheless, available studies suggest weak evidence of efficacy of PAP treatment in terms of ameliorating functional outcome and reducing stroke recurrence. Moreover, such evidence comes from few trials, often underpowered, that included patients with mild SDB and with poor adherence to PAP treatment. In fact, treatment of SA in post-stroke patients is challenging: stroke patients usually show an altered state of consciousness, cognitive impairment, and presence of nasogastric tube that compromise the adherence of ventilatory treatment. Therefore, adherence to treatment is the major issue in this peculiar population [78]. Recent studies revealed how the implementation of specific adherence programs encompassing training strategy during hospitalization [79] and telemedicine monitoring after discharge [80•] can lead to an increase CPAP adherence in post-stroke patients.

Such uncertainty of efficacy of SA treatment in post-stroke patients is reflected by diverging recommendations coming from European guidelines [13••] that endorse to screen and treat SA in post-stroke patients and American Heart Association guidelines [81••] that does not propose this approach, suggesting to enroll patients in clinical trials. Ongoing trials [82, 83] evaluating the effect of early treatment of SDB with different nocturnal ventilatory supports in acute stroke on outcome will hopefully clarify the feasibility and efficacy of such treatment in acute stroke.

Alternative treatments to PAP in stroke patients have been poorly studied (e.g., trazodone [84], swallowing interventions [85], positional therapy [86]) and are currently not recommended in the management of stroke-related SDB [13••].

## Sleep Duration, Insomnia, Hypersomnia, and Circadian Rhythms

### Risk Factors

In the last few years, sleep duration outside the recommended sleep hours has been investigated as a possible risk factor of stroke by several studies, although providing inconsistent results [87–91, 92•, 93–95]. In a recent large population-based registry, involving over one million participants, both extremely short and long sleep duration were associated with higher odds of stroke [88]. Also, in the most recent meta-analysis, which included 20 prospective cohort studies, U-shaped relationships were observed between sleep duration and stroke incidence, and mortality [8•]. However, the evidence coming from this meta-analysis points towards a slightly higher risk for long sleep than short sleep. Indeed, for short sleep duration, the relative risk of stroke was 1.33 (95% CI: 1.19–1.49), while for long sleep it was 1.71 (95% CI: 1.50–1.95) [8•]. Such difference in stroke risk was even higher in a previous meta-analysis, which found a J-shaped association between stroke and sleep duration [96]. Interestingly, a large cohort study of 79,881 Swedish participants evaluated the effect on stroke risk of single-nucleotide polymorphisms with known association to different sleep traits [97]. Such analysis revealed no association of genetic liability to short or long sleep duration with overall stroke risk but suggested a possible association between short sleep duration and increased risk of large artery stroke [97].

It is worth noting that all the studies included in the meta-analysis [8•] evaluated sleep duration as self-reported by the subjects. Conversely, in the SAVE study [90], the authors performed an instrumental estimation of sleep duration using the oximetry recording time, showing that long sleep duration was significantly associated with stroke (HR 1.79, 95% CI 1.22–2.63).

Together with short sleep duration, insomnia symptoms have been linked with increased risk of cardiovascular and cerebrovascular events, as shown by a meta-analysis of 15 studies, reporting a pooled odds ratio for the different insomnia symptoms below 1.3 [3]. However, the risk of stroke remains uncertain, since the studies included in the meta-analysis assessing stroke as outcome did not find an association between stroke and insomnia or short sleep duration, nor did a later published meta-analysis [98].

Also, an increased risk of cardiovascular comorbidity is observed in patients with narcolepsy type 1 [6], a central hypersomnia characterized by orexin deficiency. Patients with narcolepsy show lack of nocturnal blood pressure dipping, and disrupted nighttime sleep, and other comorbidities (e.g., obesity, diabetes, and mood disorders) that may concur to raise the cardiovascular risk [54]; furthermore, drugs used to manage narcoleptic symptoms may concur to increase cardiovascular risk [99].

To date, the reasons why sleep duration may influence stroke risk remain unclear. Experimental data have linked sleep deprivation to increased cardiovascular risk through several intermediate pathophysiological mechanisms involving the autonomic nervous system, endothelial function, insulin and glucose regulation, inflammation, and coagulation [100•]. Moreover, short sleep has been linked to hypercholesterolemia and increased incidence of coronary artery calcification [100•, 101]. Other biological pathways connected to short sleep are decreased secretion of melatonin [102], increased ghrelin and reduced leptin levels [103], and therefore increased appetite. Conversely, a prolonged sleep was associated with increased levels of inflammatory markers [104].

Also, circadian misalignment has been linked to increased cardiovascular risk, including stroke [7•]. The possible influence of circadian rhythms on cardiovascular disorders was supported by the observation of a circadian rhythmicity in ischemic strokes, myocardial infarction, and sudden cardiac death, all having a peak of incidence in the morning hours [105]. The circadian system influences several cardiovascular risk factors, such as circulating catecholamine levels, blood pressure, heart rate, vagal modulation, platelet aggregability, and immune responses, thus having a possible impact on stroke risk [106]. Several circadian gene polymorphisms and haplotypes have been investigated as potential genetic risk factors of stroke [107]. Genes associated with a protective role against stroke were a single-nucleotide polymorphism of CLOCK gene [108] and PER1 and PER2 genes [109]. Genome-wide association studies demonstrated an association between genetic variants of melatonin receptors 2 and the risk of metabolic disorders, such as type 2 diabetes mellitus and insulin resistance, which may in turn increase the risk of stroke [107].

Overall, evidence coming from literature points towards a slight increase of stroke risk for both short and long sleepers, as well as a possible impact of circadian misalignments on stroke risk, severity, and outcome. While multiple possible pathogenetic mechanisms linking short sleep to stroke risk have been hypothesized, the literature on the association between long sleep and stroke is scarce. Future studies should focus on the possible pathogenetic effect of sleep duration on stroke risk and clarify the respective role of short and long sleep on stroke risk by means of prospective studies with objective measures of sleep duration. As concerns circadian rhythms, starting from the observation of a circadian rhythmicity in stroke occurrence, future research should test this hypothesis on a molecular level.

### Stroke Recovery and Outcome

A growing evidence supports the knowledge that sleep disorders, pre-existent or appearing de novo, are frequent in stroke survivors and are associated with worse stroke outcomes and increased cardiocerebrovascular morbidity [110]. Notably, sleep is essential for synaptic plasticity by promoting an overall reduction in synaptic strength during slow-wave sleep and, in turn, synaptic plasticity is essential for stroke recovery. Therefore, it is presumed that poor sleep is associated to poorer stroke recovery [11].

A recent meta-analysis of PSG studies in acute ischemic stroke demonstrated that stroke patients have a poorer sleep than controls, in terms of sleep efficiency, total sleep time, and wake after sleep onset [111].

Among sleep disorders, insomnia is present in about one-third of stroke patients: in the studies assessing insomnia with validated diagnostic criteria, the pooled prevalence was 32.5% in the acute phase, and 34.8% in the subacute phase. When evaluating self-reported insomnia symptoms by means of questionnaires, the summary prevalence was 47.1% in the acute phase, and 50.4% in the subacute phase [1••]. However, it is worth to consider that insomnia is a multifactorial disorder, where a big contribution to sleep disruption is played by the hospital setting; importantly, sleep fragmentation in acute stroke is associated with an increased risk for stroke-associated delirium [112] that is, in turn, associated to poorer long-term outcome [113]. Nonetheless, studies in which insomnia was evaluated at different time points, up to 18 months from stroke onset, revealed a prevalence of insomnia symptoms near to 50% [114, 115]. Moreover, chronic post-stroke insomnia was associated with increased disability and mortality [115–117]. Even if only few studies investigated sleep complaints after the acute phase of stroke, thus limiting the generalizability of these results, the possible high risk of insomnia chronicization should be considered, and efforts should be made to limit iatrogenic sleep disruption. However, in the last years, growing evidence emerged on other factors contributing to post-stroke insomnia, further complicating the attempts to prevent this disorder. Indeed, post-stroke insomnia is often comorbid with post-stroke depression, anxiety, and fatigue with a bidirectional relationship [118•, 119]. A recent systematic review and meta-analysis on post-stroke fatigue found that depression, anxiety, and sleeping disturbances are associated with fatigue in stroke survivors, with sleeping disturbances nearly doubling the risk for post-stroke fatigue [120]. In a recent study assessing patients 1 month after stroke, poor sleep quality was independently associated with post-stroke anxiety [121]. A large, prospective study found a prevalence of post-stroke depression of 35% and of 25% at 3 and 12 months after stroke, respectively; in such cohort, sleep disturbances and fatigue were prevalent similarly to depression [122]. Interestingly, this study shows that at least 10% of patients without depression at 3 or 6 months will later develop depression at 12 months, thus suggesting the need for an active neuro-psychiatric follow-up of patients for at least 1 year after stroke.

On the other hand, hypersomnia may arise as consequence of stroke [123] affecting up to 5.6% of stroke survivors [124]. Hypersomnia moreover is a core feature of thalamic stroke [125•]. In a recent study conducted by Jaramillo et al., patients experiencing thalamic stroke showed a reduction of overnight slow wave slope changes suggesting an impaired thalamic-dependent synaptic renormalization, and therefore, an impaired recovery [125•]. Moreover, post-stroke hypersomnia in stroke patients is linked to poorer functional outcome and to an increase risk to go in a nursing home, suggesting an impaired sleep-dependent recovery of stroke patients [124].

The frequent co-existence of sleep disorders, depression, anxiety, and fatigue after stroke implies the need for interventions which could possibly target all these aspects. A 6-week therapy with modafinil, a wakefulness-promoting agent, was investigated for treatment of post-stroke fatigue persisting 3 months or more after stroke, showing a benefit on fatigue and quality of life [126]. Post-stroke depression is a clinical entity which is poorly responsive to pharmacological approaches [127, 128]. However, a recently published meta-analysis showed that SSRIs are effective in treating post-stroke depression and anxiety, and improving post-stroke recovery in terms of motor function, cognitive function, and dependence [129•]. Treatment of post-stroke insomnia is challenging as well: indeed, GABA agonists may have detrimental effects on stroke recovery; they have not been systematically evaluated in patients with post-stroke insomnia and therefore no recommendations can be made on their use [13••]. Apart from pharmacotherapy, other approaches have been investigated for post-stroke depression and insomnia, such as psychotherapy, bright light therapy, and acupuncture, with some evidence of a benefit on sleep parameters, daytime sleepiness, fatigue, mood, and quality of life [130–132]. In a recent randomized controlled trial, ischemic stroke patients with comorbid depression and insomnia were randomized to receive bright-light therapy and escitalopram or escitalopram alone. Compared to monotherapy, polytherapy significantly improved depressive symptoms and sleep complaints [133].

Sleep–wake cycle is impacted after stroke as well, as shown by studies involving actigraphy recordings and chronotype questionnaires [118•]. Moreover, circadian rhythm’s dysfunction is supported by the finding of reduced melatonin levels in patients with acute stroke [118•]. Therefore, the current pre-clinical research is oriented towards melatonin supplementation in experimental models of ischemic stroke. A recent study on mice found that daily melatonin administration during the subacute phase of stroke ameliorated stroke-induced sleep disturbances and resulted in reduction of infarct volume [134]. However, the efficacy of melatonin supplementation in human subjects with acute stroke has not been systematically evaluated, limiting the translationality of these results. Moreover, findings coming both from pre-clinical and clinical models of stroke suggest an impact of circadian rhythms also on the outcome of stroke. Indeed, a recent experimental study on mice suggested that stroke onset at different sleep–wake time points has an impact on stroke severity and outcome, which were worse for stroke occurring during sleep, compared to those occurring at wake [135]. In a multicenter study, including more than 17,000 patients, night-onset strokes, compared with day-onset strokes, were associated with worse presenting neurologic severity, more frequent early neurological deterioration, and worse functional outcome [136]. However, such association between sleep-onset and worse stroke outcome may be at least partially explained by a delayed recognition of stroke symptoms.

Overall, given the bidirectionality of relationship between sleep and mood disorders after stroke, treatment of post-stroke sleep and sleep–wake cycle disorders, depression, and fatigue should encounter a multi-component approach with target on sleep–wake cycle improvement, appropriate neuro-rehabilitation, and psychotherapy.

## Restless Legs Syndrome, Periodic Limb Movement of Sleep, and Rem Sleep Behavior Disorder

RLS is a disturbance characterized by an unpleasant sensation in the legs and an irresistible urge to move them, occurring typically in the evening hours. RLS is often associated with PLMS, a condition with repetitive limb movements that occurring during sleep and may cause sleep disruption.

RBD is a REM-related disorder characterized by unpleasant dreams and vigorous motor behaviors in which the patients seem to be enacting their dreams. RBD is an early prodromal sign of the α-synucleinopathies.

Such disturbances have been investigated as risk factors for stroke and as consequence of stroke.

### Risk Factors

In the last years, RLS and PLMS have been suggested as possible risk factors for stroke by different observational studies. Nevertheless, two systematic reviews and meta-analysis on RLS did not provide any evidence for an increased risk of stroke in patients with RLS [4, 137]. Conversely, a systematic review and meta-analysis on PLMS concluded that PLMS is associated with a mild increased risk for stroke (OR 1.267; 95% CI 1.040–1.543) [138]. However, the results of this meta-analysis should be taken cautiously since the five studies included did not control for other stroke-associated factors, such as presence of anti-thrombotic therapy, anti-hypertensive medications, strict diabetes mellitus management, smoking, and OSA. Therefore, further studies controlling for other stroke-related risk factors are needed to assess the true impact of PLMS on the risk of stroke. Also, long-term, prospective studies, controlling for potential confounders, are needed to rule out the hypothesized association between RLS and cardio-cerebrovascular morbidity.

RBD is characterized by early impairment of autonomic nervous system and of a non-dipping profile of blood pressure that may concur to increase the risk of cerebrovascular accidents [139]. However, to date, there is no convincing evidence of an increased risk of stroke in patients with such disorder; only a recent study suggests a higher risk of developing both hemorrhagic and ischemic stroke in patients with probable RBD, not confirmed by means of PSG [5].

### Consequence of Stroke and Role on Stroke Recovery

In the last years, research on stroke-related RLS has focused on its incidence and on the identification of possible connections with the anatomy of stroke lesions. Data emerging from the most recent literature are not homogenous concerning stroke-related RLS incidence, which ranges between 2.3 and 15.1% [140•]. The most common sites of stroke lesions involved in stroke-related RLS have been identified in basal ganglia, corona radiata, thalamus, and brainstem, especially pons [140•]. In a recent prospective study, stroke lesions involving the body of caudate nucleus, the lenticulo-capsule, and corona radiata were significantly more frequent in the cohort of patients with stroke-related RLS compared with controls [141].

An element of uncertainty is whether the different clinical presentations of the disorder, which is more often unilateral, usually located in the paralyzed lower limb [142], may hide different pathogenetic mechanisms. Indeed, the pathogenesis of bilateral stroke-related RLS is yet to be determined, since the anatomical location of stroke can only partially explain the symptoms [140•]. A recent prospective study found bilateral symmetrical stroke-related RLS in 13/16 patients, even if all patients had unilateral stroke [143]. The lenticulostriate area was involved in eight patients, being either left-sided or right-sided, whereas seven patients had ventral brainstem stroke. Interestingly, a hyperdopaminergic tone in the putamen ipsilateral to the infarct was observed in all patients, except in one [143]. From a genetic point-of-view, direct evidence is rather scarce regarding the association between genes regulating dopaminergic neurotransmission, iron metabolism, and cardiovascular risk [107]. Some studies have argued that RLS is associated with a less favorable outcome of stroke [144, 145]; however, larger studies are needed to confirm these data [13••].

For what concerns PLMS, data on its prevalence after stroke are inconclusive [13••]. A large, prospective, polysomnographic study reported a similar frequency of PLMS in 169 stroke patients compared with 162 controls, both in the acute and subacute phase [146]. A meta-analysis, including 158 PLMS patients with stroke and 88 PLMS controls without stroke, revealed a significantly higher periodic limb movement index in patients with stroke, compared with controls [138]. However, studies investigating the pathophysiology of stroke-related PLMS are warranted to clarify the finding of a possible worse PLMS severity after stroke. Moreover, future studies with larger cohorts are needed to investigate its prevalence and its impact on the outcome of stroke.

Also, RBD may arise de novo as a consequence of brainstem stroke affecting nuclei involved in the regulation of REM sleep [147]. The pathogenic mechanisms are not clear; a recent study conducted on patients with brainstem stroke observed a preserved REM atonia of both phasic and tonic activities and only a reduction of the total time spent in REM [148].

To the present, data on the incidence of movement disorders of sleep and RBD after stroke, as well as their influence on further stroke risk and recovery, are inconclusive.

## Conclusion

Despite substantial medical literature indicates a pivotal role of sleep and its disorders from pre-stroke up to chronic stroke phase, to date, poorness is known on pathogenic mechanisms and optimal management of sleep disorders in stroke population (see Table 1). From this point of view, rigorous and large trials are warranted in order to elucidate the role and the management of each specific sleep disorders in patients with stroke.

**Table 1:** The role of sleep disorders in pre-stroke, acute stroke and chronic stroke phases

	Risk factors	Acute stroke	Chronic Stroke
OSA	Indipendent risk factor	Highly prevalent May be aggravated in acute stroke	Associated with stroke recurrence and worse outcome
CSA	No definite role	The exact prevalence is not clear May appear de novo after stroke Possible predictor of mortality	May ameliorate in chronic phase
Insomnia	Possible risk factor	Sleep fragmentation is a risk factor for stroke-associated delirium	Highly prevalent Poor sleep quality is associated with worse outcome
Hypersomnia	Possible risk factor	Core feature of bilateral thalamic stroke	Possible association with worse outcome May overlap with fatigue in post-stroke syndrome
Circadian rhythm	Circadian misalignment is a possible cerebrovascular risk factor Polymorphisms of circadian-regulated genes may have a protective role	Impaired in acute stroke	Night-time stroke is associated with worse neurological outcome
RLS and PLMS	Possible risk factor	May appear de novo after stroke involving basal ganglia and brainstem	Possible association with worse outcome
RBD	Possible risk factor	May appear de novo after brainstem stroke	Not investigated

Even more important, sleep is poorly investigated by stroke physicians in the standard clinical practice outside research programs. Current knowledge supports that sleep assessment should promptly enter in the routinary stroke care.
